# Uterine Microbiota: Residents, Tourists, or Invaders?

**DOI:** 10.3389/fimmu.2018.00208

**Published:** 2018-03-02

**Authors:** James M. Baker, Dana M. Chase, Melissa M. Herbst-Kralovetz

**Affiliations:** ^1^Department of Basic Medical Sciences, College of Medicine-Phoenix, University of Arizona, Phoenix, AZ, United States; ^2^Department of Biology and Biochemistry, University of Bath, Bath, United Kingdom; ^3^Arizona Oncology (US Oncology Network), University of Arizona College of Medicine, Creighton University School of Medicine at St. Joseph’s Hospital, Phoenix, AZ, United States; ^4^Department of Obstetrics and Gynecology, College of Medicine-Phoenix, University of Arizona, Phoenix, AZ, United States

**Keywords:** endometrium, microbiome, host–microbe interactions, gynecologic and reproductive health, inflammation, infertility, endometrial cancer, pathophysiology

## Abstract

Uterine microbiota have been reported under various conditions and populations; however, it is uncertain the level to which these bacteria are residents that maintain homeostasis, tourists that are readily eliminated or invaders that contribute to human disease. This review provides a historical timeline and summarizes the current status of this topic with the aim of promoting research priorities and discussion on this controversial topic. Discrepancies exist in current reports of uterine microbiota and are critically reviewed and examined. Established and putative routes of bacterial seeding of the human uterus and interactions with distal mucosal sites are discussed. Based upon the current literature, we highlight the need for additional robust clinical and translational studies in this area. In addition, we discuss the necessity for investigating host–microbiota interactions and the physiologic and functional impact of these microbiota on the local endometrial microenvironment as these mechanisms may influence poor reproductive, obstetric, and gynecologic health outcomes and sequelae.

## Background

**Figure 1:**
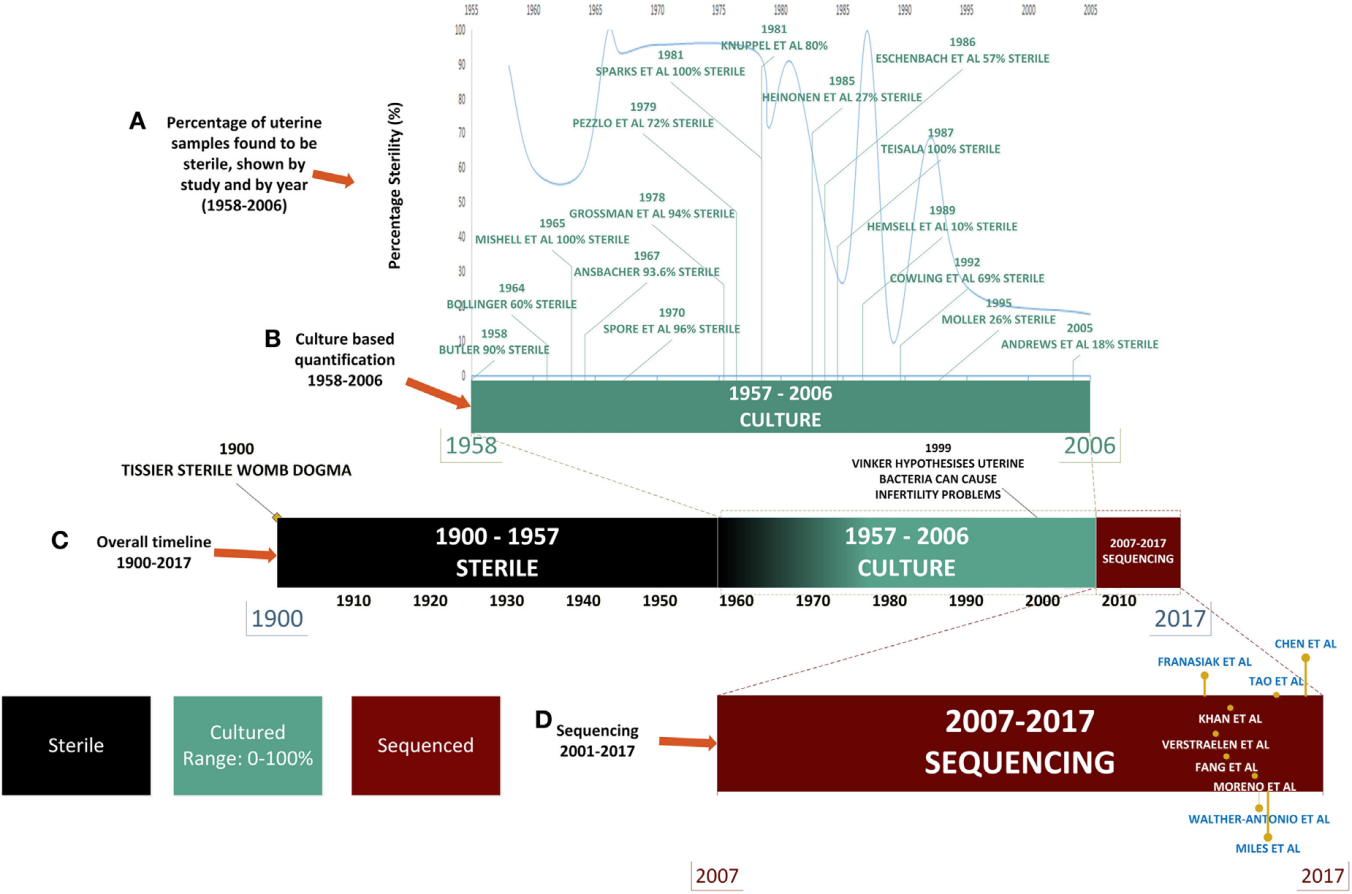
Timeline of uterine microbiota reports in the literature. Color legend: black denotes time periods in which the uterus was considered sterile, green indicates the time period during which culture-based techniques were used, and red indicates the uses of high-throughput sequencing techniques. **(A)** From 1958 to 2006: literature detailing culture-based methods of quantification of the uterine microbiome (shown in green). **(B)** The decline in the perceived sterility of the endometrial microbiome according to literature reported over time. **(C)** Overall timeline from 1900 to 2017 [from assumption of sterility of uterine microbiome to detailed quantification of bacterial species through next-generation sequencing (NGS)]. **(D)** NGS: 2007–2017 (shown in red) uterine microbiome literature that has been published to date utilizing this technology.

**Figure 2:**
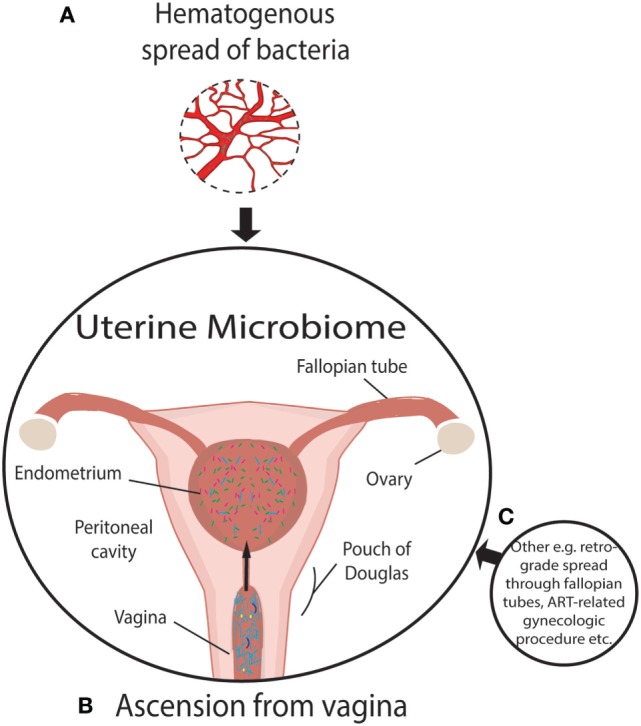
Established and putative bacterial transmission routes between uterine microbiome and distal sites. **(A)** Putative hematogenous spread of bacteria emanating from the gut and oral microbiome or other means of circulation of bacteria through the blood. Viability has been demonstrated to be conserved during translocation through blood with intracellular dormancy being an example of how bacteria remain viable in blood. **(B)** Ascension of bacteria through the cervix has been well established and is a likely source of bacterial transmission. **(C)** Transmission of bacteria through routes, other than those illustrated, include assisted reproductive technology-related gynecologic procedures whereby bacteria from the vaginal microbiome are introduced to the uterus, such as oocyte retrieval. Other routes of colonization may exist beyond hematogenous spread. The insertion or removal of intrauterine devices may introduce bacteria into the uterus as well as potentially aid in ascension through the “tails” that extend from the uterus through the endocervical canal.

For almost a century and based on the work of Henry Tissier in 1900, consensus was that a healthy uterine cavity is sterile (Figure 1) (1). This sterility was hypothesized to be maintained by the cervical plug, which was compared with the “Colossus of Rhodes” in providing an impermeable barrier to bacterial ascension from the vagina (2). This assumption was challenged by multiple reports in the mid to late 1980s, using culture-dependent methods, of uterine-dwelling bacteria even in healthy asymptomatic women (Figure 1) (3–6). Furthermore, the cervical mucus plug has been shown to not be entirely impermeable to bacterial ascension from vaginal bacteria (7, 8). It was also shown that in a non-pregnant state, particles can translocate from the vagina to the uterus through the cervical canal within minutes during the follicular and luteal phases of the cycle (9). The naturally occurring uterine peristaltic pump aids in sperm transport from the cervical canal to the uterus, and these peristaltic contractions have been shown to move macrospheres from the canal into the uterus and other areas of the upper female reproductive tract (FRT), and therefore may play a role in seeding the uterus with bacteria (8). The follicular phase of the menstrual cycle has been shown to be associated with an increased frequency of uterine contractions (10). Uterine conditions may also promote bacterial seeding of the uterus through hyper- and dysregulation of uterine contractions (10). In addition, it was argued that the position of the uterus in such close proximity to a consistently colonized site such as the vagina would make some movement of bacteria to the uterine cavity inevitable. The presence of a uterine microbiome has been reported in animal models, most notably in cows, where the impact of the uterine microbiome on fertility is a key contemporary research question (11–15). Specific bacterial species have shown a tendency for colonizing the uterus, such as *Fusobacterium*, which has been found in both mice and cow uteri (16). Colonization with this particular bacterium in mice has been shown to be transmitted through the hematogenous route (Figure 2) (17). Hematogenous (through the bloodstream) spread of bacteria through either oral (18) or the gut route (19) allows bacteria from mucosal sites such as the oral cavity and the gastrointestinal tract to colonize distal mucosal sites and occurs during epithelial barrier breach (e.g., gingivitis and leaky gut) (17, 20–23). However, other sources of uterine microbiota seeding may include inadvertent bacterial transmission of vaginal bacteria into the uterus through assisted reproductive technology (ART)-related procedures or during placement of intrauterine contraceptive devices (Figure 2) (24, 25).

Later the assumption became that any detection of bacteria in the uterus was the result of ascension from the lactobacilli (LB)-rich vaginal microbiome. The presence of bacteria in the uterus has been associated as causative agents in adverse conditions such as recurrent abortion and preterm labor (23, 26). An enormous hurdle to twentieth century research in this area was the necessity to culture bacterial specimens for analysis, which severely limited quantification and resolution of bacterial communities, leading to low and inconsistent bacterial yields (Figure 1). This is particularly important when one considers that the uterus is a low abundance site (27, 28). Estimations of uterine bacteria load are estimated between 100 and 10,000 times less bacteria than the vaginal microbiome (27, 28).

The advent of next-generation sequencing (NGS) technologies in 2007 has enabled a far more global assessment of bacterial composition of the uterus than could be measured solely with culture-dependent methods. Furthermore, culture studies focused on the ability to culture a finite variety live bacteria, whereas sequencing technologies enabled the identification of the full range of uterine bacteria (26). Indeed, it is now appreciated that only approximately 1% of bacteria are culturable (29–31). NGS has enabled species-level quantification utilizing the variable (V) regions of the 16S rRNA gene, potentially allowing for the determination of the full scope of uterine microbiome signatures in both healthy and diseased hosts.

The purpose of this review is to provide a comprehensive summary of the uterine microbiome literature to date, focusing on detailing studies of “healthy” bacterial residents to bacterial tourists or invaders that are present in particular diseased states. This review will consider the limitations of NGS at a low abundance site such as the uterus and discuss key methodical considerations pertaining to sampling the uterus and unique challenges to collecting these patient specimens. In addition, we identify gaps in the area of uterine microbiota research that will provide exciting research opportunities for future studies. The ascertainment of uterine microbiota signatures in various states of health and disease could potentially lead to effective clinical interventions for a variety of conditions and have a positive impact on obstetric and gynecologic health.

## Residents, Tourists, or Invaders: Defining Uterine Microbiota

The current NGS literature on the uterine microbiome has provided provocative glimpses into the putative role of the uterine microbiota in multiple disease states and the potential impact on women’s health. It is important, however, to take into consideration the limitations of the studies to date. These considerations include reagent controls, subject cohort size and patient demographics, and sample collection, sequencing methods, and downstream analyses.

The possibility of sample contamination is a significant hurdle to ascertaining whether uterine bacteria are residents, tourists, or invaders due to the low abundance of bacteria in the uterus (32). Contamination may contribute to a larger percentage of microbiota enriched and reported at low abundance sites. The placenta and lung are also low abundance sites that have divergent reports in the literature with some groups detecting a distinctive placental microbiome (33), while others find it to be indistinguishable from negative controls (34). These studies illustrate the difficulty in distinguishing low abundance sites from false positives and the requirement for larger studies and need for robust controls.

Contamination controls, when reported, did not fully describe how these control samples and data were accounted for during downstream analysis of samples. Walther-António et al. reported contamination in 9 of their 14 negative controls, yet it was not described how these controls were accounted for in the sample analysis (35). This is fairly typical of the current literature, whereby a detailed description of control samples are not clearly described. However, Chen et al. included more rigorous controls and reported inclusion of both extraction controls and PCR controls (27). This group also included additional controls when culturing peritoneal fluid, using diluent controls and swabs taken from sterilized skin of the patient as well as swabs of the doctor gloves (27). Most uterine sampling is performed transcervically, which makes it difficult to avoid cross-contamination with the cervical microbiota (see Table 1). In addition, uterine manipulators and cervical dilators may further contribute to cross-contamination from the cervix if used during hysterectomy procedures; however, studies rarely report if these instruments were used. In most studies in this review (Table 1), precautions were taken to limit contamination through methods such as vaginal disinfection, which coupled with careful sampling, reduces contamination. Nevertheless, rigorous application of contamination controls and detailed descriptions of clinical procedures are an important aspect of future research (32). Future studies may consider including negative controls that can measure reagent contamination that can be subtracted from the experimental samples (36).

**Table 1:** Patient demographics, study design, and profile results of current literature describing next-generation sequencing studies of uterine microbiota in chronological order.

	Patient population and objective	Patient cohort (subjects and age)	Race and/or ethnicity	BMI	Sample type analyzed and pH collected	Procedures to avoid contamination from vagina or cervix?	Contamination controls and type	Sequencing platform and variable region	Top identified phyla	Summary of findings
Mitchell et al. (28)	Patient population: women undergoing hysterectomy for non-cancer indicationsObjective: to evaluate the presence of vaginal bacterial species in the uterus	58 subjects Average age: 43 No use of antibiotics within the last 30 days	White: 79% African American: 10% Hispanic: 7% Declined to answer: 3%	NR	Endometrial swabs from excised uterus Vaginal swabs collected before hysterectomy pH: NR	Specimens were collected only if the surgeon was able to complete the procedure using a noninvasive vaginal fornix delineator (Colpo-Probe; Cooper Surgical, Trumbull, CT, USA) or a vaginal sponge stick rather than an intracervical manipulator	NR	qPCR for 12 species	Firmicutes Bacteroidetes	- 95% of subjects had upper genital tract colonization The three most common species in uterus were *L. iners, Prevotella* spp., and *L. crispatus* - No significant difference in soluble markers of inflammation in endometrial swabs between women with (a) no bacteria, (b) only *Lactobacillus* species, or (c) detectable non-*Lactobacillus* species pH: NR

Franasiak et al. (48)	Patient population: women undergoing IVF^a^ Objective: to assess the impact of microbiome obtained from IVF catheter tip at the time of embryo transfer on pregnancy outcome following IVF	33 subjects Average age: 35.9 Antibiotics: NR	Caucasian: 79% Asian: 15% African American: 3% Hispanic: 3%	NR	Distal 5 mm of IVF catheter tip pH: NR	Formable outer sheath advanced under ultrasound guidance	Positive controls utilizing *Escherichia coli* along with negative controls were run to detect contamination from reagents	The Ion 16S Metagenomics Kit (V2–4–8 and V3–6, 7–9)	Firmicutes Proteobacteria	- *Lactobacillus* was the top genus found on the IVF catheter tip in both successful and unsuccessful IVF outcomes - *Flavobacterium* was the second most prevalent genus found across both groups - There were no characteristic differences in microbiomes between successful and unsuccessful IVF groups pH: NR

Verstraelen et al. (50)	Patient population: women with various reproductive conditions (recurrent implantation failure, recurrent pregnancy loss, or both) but no uterine abnormalities and a diverse medical history Objective: to determine the presence of a uterine microbiome in non-pregnant women	19 subjects Median age: 32 No perioperative antibiotic treatment	White: 100%	NR	Tao BrushTM IUMC Endometrial Sampler pH: NR	Cervical surface and external os were thoroughly rinsed with an aqueous 0.5% chlorhexidine gluconate solution (antiseptic and disinfectant). Tao BrushTM IUMC Endometrial Sampler protected by a plastic covering sheath laterally and by a small plastic bead on top to protect the brush on all sites from contamination during passage through the vaginal lumen and endocervical canal	NR	Illumina (V1–V2)	Bacteroidetes	90% of the subjects had uterine microbiomes in which *Bacteroides xylanisolvens, Bacteroides thetaiotaomicron, Bacteroides fragilis*, and an undetermined *Pelomonas* taxon made up over a third of the total pH: NR

Fang et al. (49)	Patient population: women with EP and “healthy” asymptomatic women with partners with MFI Objective: to determine the difference between uterine microbiota composition between EP, EP + CE, and “healthy” asymptomatic controls	30 subjects Average age: H: 30.90 EP + CE: 35.2 EP: 34.4 No antibiotic use within last 3 weeks	NR: study conducted in China	H: 21.04 ± 1.03 EP: 20.47 ± 0.67 EP + CE: 21.29 ± 0.99	Vaginal swabs and endometrial swabs collected pH: NR	Vaginal and cervical canal disinfection Endometrial swabs with sleeves	NR	Illumina (V4)	Proteobacteria Firmicutes	- Subjects with EP and EP/CE had microbiomes with much higher proportions of Firmicutes than healthy subjects - At the genus level, *Lactobacillus, Gardnerella, Bifidobacterium, Streptococcus*, and *Alteromonas* were significantly higher in the healthy group compared with either the EP or the EP/CE group - *Enterobacter* and *Sphingomonas* were found at lower proportions and *Prevotella* at a higher proportion in the EP/CE group pH: NR

Khan et al. (53)	Patient population: healthy asymptomatic women operated on for dermoid cyst/serous cyst adenoma/mucinous cyst adenoma or for uterine myoma and women with endometriosis. Both groups were further divided into GnRHa treated and GnRHa-untreated Objective: to assess the impact of endometriosis and/or GnRHa treatment on the intrauterine microbiome	32 subjects Average age: Control GnRHa−: 33.6 Control GnRHa+: 42.1 Endometriosis GnRHa−: 35.7 Endometriosis GnRHa+: 37.5 Antibiotics: NR	NR: study conducted in Japan	NR	Seed swabs were used to collect endometrial samplesCystic fluid was collected during laparoscopy pH: NR	Seed swab was inserted under visual control into the uterine cavity taking care to avoid any contact with vaginal walls	NR	Illumina (not specified)	Firmicutes Proteobacteria	- *Lactobacillaceae* were significantly decreased in women with endometriosis being treated with GnRHa compared with without endometriosis but were also treated with GnRHa - *Streptococcaceae, Staphylococcaceae*, and *Enterobacteriaceae* were significantly increased in women treated with GnRHa compared with women without endometriosis but were also treated with GnRHa pH: NR

Moreno et al. (47)	Patient population: women undergoing IVF 19–29 kg/m^2^ whom had at least one good-quality embryo transferred but had not used antibiotics within the last month before the study Objective: to determine the impact of the uterine microbiome obtained from IVF catheter tip at the time of embryo transfer, and its hormonal regulation by collected endometrial fluid at 2 days after luteinizing hormone surge as well as 7 days after, on reproductive out in those undergoing IVF	Subject numbers: Impact of uterine microbiome on reproductive success: 35 Impact of hormonal regulation on uterine microbiome: 22 Comparison of vaginal microbiome and uterine microbiome: 13 Average age: LD: 40.06 NLD: 39.00 No antibiotics or probiotics used within the last month	NR: study conducted in Spain as part of the ovum donation program	LD: 24.18 ± 5.18 NLD: 22.45 ± 4.02	Endometrial fluid collected with catheter inserted transcervically pH: endometrial	To prevent contamination by cervical mucus during catheter removal, suction was dropped at the entrance of internal cervical oss (ICO), and cervical mucus was also aspirated before EF aspiration	NR	454 Pyrosequencing (V3–5) Firmicutes Actinobacteria		- Uterine microbiota did not differ at two timepoints in the hormonal cycle - The presence of a non-*Lactobacillus*-dominated uterine microbiota in a receptive endometrium was associated with significant decreases in implantation, pregnancy, ongoing pregnancy, and live birth rates pH: endometrial pH not associated with microbiota composition or reproductive outcome

Walther-António et al. (35)	Patient population: women undergoing hysterectomy for either benign uterine conditions, endometrial hyperplasia or endometrial cancer Objective: to determine uterine microbiome in patients with and without endometrial cancer	31 subjects Median age: Benign: 44.5 Cancer: 64 Hyperplasia: 54 No antibiotic 2 weeks prior	Caucasian: 100%	Median: Benign: 26.6 Cancer: 32.1 Hyperplasia: 35.4	Uterine, fallopian tube, ovary, and peritoneal swabs following hysterectomy. The uterus, vagina and cervix also had scrapes taken. Urine and stool samples were also taken pH: vaginal		A total of 14 controls were performed, with five of them not retrieving any sequence readsA Petri dishwith Lysogeny broth was kept open on the grossing station during sample collection to detect any possible airborne contamination of the specimen (findings NR)	Illumina (V3–5) Proteobacteria Bacteriodetes		- Vaginal, cervical, fallopian tube, and ovary microbiomes are significantly correlated within an individual - *Atopobium vaginae* and a *Porphyromonas* sp. in the gynecologic tract were statistically associated with endometrial cancer pH: high vaginal pH associated with endometrial cancer

Miles et al. (51)	Patient population: women undergoing hysterectomy and salpingo-oopherectomy for a variety of conditions Objective: to determine the microbial compositions at various sites in the female reproductive tract (FRT) and to what extent it varies between patients	10 subjects Average age: 50.6 No antibiotic treatment within the last 30 days. All patients received antibiotics 30 min before surgery	NR	NR	Endometrial, vaginal, cervical, myometrial, fallopian tube, and ovarian swabs taken post-hysterectomy pH: NR		Quality assurance and control of the reactions were performed with both positive and negative control samples to ensure fidelity of the reagents and lack of contamination	454 Pyrosequencing (V1–3)	Firmicutes Proteobacteria	- At a phylum level, Firmicutes were highly abundant - At a genus level, *Lactobacillus* were highly abundant - Bacterial profiles were highly related across all samples and across all patients pH: NR

Tao et al. (52)	Patient population: women undergoing IVF^a^ Objective: to determine the microbiome obtained from IVF catheter tip during embryo transfer and to assess the limit of accurate quantification of microbiota	70 subjects Average age: 36.2 Antibiotics: NR	Caucasian: 61% Asian: 17% African American: 1.4% Hispanic: 5.6% Unknown: 15%	NR	Distal 5 mm of IVF catheter tip pH: NR	Formable outer sheath advanced under ultrasound guidance	Positive controls at varying concentrations for both single species and polymicrobial samples were used to validate the detection of low abundance bacteria. A negative control was also included	Illumina (V4)	Firmicutes	- Firmicutes were highly abundant from IVF catheter tip - At a genus level, *Lactobacillus* were highly abundant from the IVF catheter tip - *Lactobacillus* were detected in all patients sampled along with other vaginal bacteria pH: NR

Chen et al. (27)	Patient population: women operated for conditions not known to involve infectionObjective: to determine the microbiota along the FRT and its association with menstrual cycle, adenomyosis and endometriosis	110 subjects Age: NR No recent use of antibiotics	Asian: 100%	NR	Nylon flocked swabs used to sample: lower third of vagina, posterior fornix, cervical mucus, endometrium, left fallopian tube, and right fallopian tube. Peritoneal fluid was sampled after sterile saline was injected into the peritoneal cavity		Negative diluent controls used: sterile PBS, sterile physiological saline, dry sterile swabs rubbed on preoperative skin, and dry sterile swabs rubbed on surgeon’s gloved fingers. The controls were then cultured on PYG agarPeritoneal fluid was collected from 15 women and were cultured on PYG agar	Ion Torrent Personal Genome Machine system (V5–V4)	Firmicutes	- Unique microbiota compositions were found to exist in cervical canal, uterus, fallopian tubes and peritoneal Fluid which differed from the vagina - Microbiota was also found to correlate with endometriosis and stage in the menstrual cycle - Uterine microbiome shown to be culturable in 5 out 15 subjects

*IVF, in vitro fertilization; EP, endometrial polyps; CE, chronic endometritis; MFI, male factor infertility; NR, not reported; GnRHa, gonadotropin-releasing hormone agonist; LD, Lactobacillus dominant; NLD, non-Lactobacillus dominant*.

*^a^No report of any prevailing medical conditions which may modulate microbiota*.

Subject cohort size is another key limitation of the field, due in part to the difficulty of enrolling patients and the technical challenges in obtaining uterine samples. Six of the nine studies focused on in this review had a cohort of ≤35. This small sample size severely reduces the statistical power of the studies (Table 1). A related issue is the lack of ethnic diversity across these small-scale studies. It is already well documented that bacterial vaginosis (BV) differs significantly in Caucasian women compared with other racial and ethnic groups (37). It is therefore likely that the same would be true of the uterine microbiota as suggested by a recent abstract (38). If the uterine microbiome varies with ethnicity this may have potential impact on risk factors associated with gynecologic and reproductive sequelae, but should also account for differences in socioeconomic factors and environment (e.g., diet) (39, 40). Clearly, larger and more inclusive studies are needed.

One seemingly unavoidable limitation of this line of research is the lack of healthy controls which results from the fact that healthy excised uteri are rarely obtained. All hysterectomies were carried out due to an underlying benign or non-neoplastic condition or a symptomatic condition such as fibroids. However, the issue of appropriate controls also extends to studies assessing the microbiota of *in vitro* fertilization (IVF) patients; even though there may not be frank disease as such, these women can still not be considered healthy controls due to infertility. IVF studies where the inclusion criterion is restricted to male factor infertility provide a better control population (Table 1). Other factors that affect “healthy” controls include antibiotic usage and collection of a detailed medical history and use of clear exclusion criteria. For example, women with an intrauterine device (IUD) should be excluded due to their potential impact on uterine colonization, unless this is related to the question being addressed. Mitchell et al. were the only group that excluded IUD users despite IUDs being known to harbor bacteria and aid in uterine colonization (25, 28).

The specific 16S rRNA gene V region primers used by studies in this area (shown in Table 1) are a potential cause of incongruence as certain 16S rRNA gene V regions have been shown to over- or underrepresent certain taxa (41, 42). In addition to the choice of 16S rRNA gene V region primers, DNA extraction methods and operational taxonomic unit classification have also been identified as potential sources of variation in microbiome studies (43). Adoption of standardized methodology in these areas would greatly facilitate comparisons across studies.

While NGS provides a useful tool in bacterial quantification, it only quantifies bacterial the 16S rRNA gene, it does not represent viability. As pointed out in the recent review by Perez-Muñoz et al., this is a significant limitation in the field (32). While bacteria have been cultured from the uterus in numerous studies since the 1950s and in the recent report by Chen et al. there is still a question as to whether these bacteria quantified by NGS represent viable bacteria.

The ability for germ-free mice to be generated provides some evidence against a resident uterine microbiome as the process involves the removal of the pregnant uterus from conventional mice, placing in a germicidal bath and then transferring them to a germ-free mother. However, low abundance uterine microbiota may be removed as a result of the germicidal bath. While not the focus of this review, the bacterial seeding of the uterus has important ramifications related to the highly debated topic of maternal–fetal transfer of microbiota and postnatal health (32, 44). The presence or absence of a placental microbiome remains a controversial topic as it relates to maternal–fetal transfer of the microbiome and is beyond the scope of this review (21, 45). Currently, the data available suggest that maternal gut microbiota impacts fetal health outcomes (46). Whether this is through interaction of bacteria and the placenta/amniotic fluid/meconium directly, or whether the interaction is through microbial products or metabolites, remains to be fully elucidated and may not be mutually exclusive.

While there are certainly limitations in the studies to date, the current literature demonstrates significant changes in microbiota compositions related to various disease states, rates of IVF success, and risk for endometrial cancer. These studies have provided a starting point for future studies in uterine microbiome research and to expand our fundamental understanding of this emerging aspect of human health.

## Residents: Uterine Microbiota in “Healthy” Asymptomatic Women

This review mainly focuses on the negative consequences of the presence of uterine microbiota due to inherent difficulties in sampling the uterus in healthy women; however, clearly the maintenance of homeostasis is also important if a “normal” resident microbiome in the uterus is defined. The uterine microbiota reported in healthy subjects, as defined by NGS, varies greatly throughout the nine reports that exist to date (Table 1). With little consistency extending all the way up to the phyla level, it is currently difficult to define a consensus “healthy” or “core” uterine microbiota. However, certain generalizations can be made from the existing data. The most abundant bacteria consistently belong to the following phyla: Firmicutes, Bacteriodetes, Proteobacteria, and Actinobacteria (27, 35, 47–53).

Within the Firmicutes, the genus *Lactobacillus* is a very prominent component in the majority of the uterine microbiome studies and is a consistent finding among reports to date (Table 1) (28, 47–49, 51, 52). Again, however, comparison of the relative abundance (or even absence) of *Lactobacillus* between sequencing reports highlights the inconsistency among reports and warrants further investigation. For example, Fang et al. reported higher levels of *Lactobacillus* in diseased groups of women with endometrial polyps (EP) or in women with EP and chronic endometritis (EP + CE) (38.64 and 33.21%, respectively) compared with healthy controls (6.17%) (49). By contrast, Moreno et al. reported that high levels of *Lactobacillus* (>90% as defined by the group) are significantly associated with increased reproductive success in women undergoing IVF, although whether only certain (undefined) *Lactobacillus* species may be capable of conferring this benefit is not clear from this study (47).

**Figure 3:**
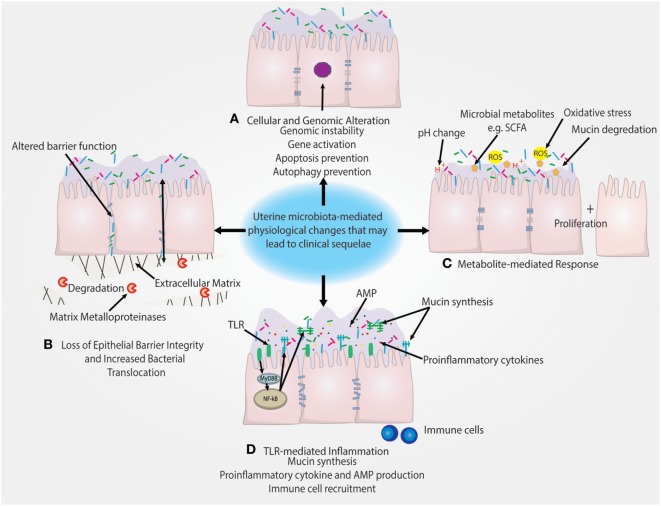
Putative pathophysiological impact of uterine microbiome on the endometrial epithelium. **(A)** Presence of uterine microbiota may impact the genomic stability of uterine epithelia through modulation of transcription factors and other genomic and epigenetic alterations. This may subsequently lead to the prevention of autophagy. **(B)** Downregulation of cell–cell junction expression is a key method of epithelial barrier breach and allows for the movement of bacteria in between epithelial cells. Similarly, the degradation of the extracellular matrix by matrix metalloproteinases also disrupts epithelial barrier integrity. **(C)** Microbial-secreted metabolites such as short-chain fatty acids (SCFAs) can encourage the growth of specific species and suppress growth of other bacteria. Reactive oxygen species (ROS) and changes in the pH of the uterine microenvironment may also drive disease. **(D)** Inflammation triggered by TLR activation and subsequent pro-inflammatory pathways can recruit immune cells and lead to the secretion of antimicrobial peptides (AMPs), which leads to the depletion of bacterial abundance. TLR-mediated signaling can also regulate mucin synthesis of both membrane-associated and secreted mucins that may impact colonization.

Notably, *Lactobacillus* dominance is generally considered to be a predictor of vaginal health (54, 55). However, increased level of *Lactobacillus* may act as a risk factor or marker for EP + CE through a breach in the cervical barrier that subsequently allows for ascension of *Lactobacillus* from the vagina to the uterus. The increased reproductive success in women with high levels of *Lactobacillus* may simply reflect the composition of the vaginal microbiome at time of IVF ET (56). It is also important to consider that assessing the uterine microbiota by catheter tip analysis may not be true representation of the bacteria in the uterus. The sampling and surface area that the catheter tip assesses is dramatically smaller compared with swabbing the uterus. This likely decreases the quantity of bacteria obtained and therefore increases the impact of contaminants either from the cervicovaginal environment or the reagents. However, the uterine microenvironment is unique from other mucosal sites in that it serves as the starting point for embryo implantation and placentation and is tightly regulated by female sex hormones. Therefore, unlike the vagina, *Lactobacillus* may not be a predictor of uterine health and could have pathophysiological consequences following ascension from the vagina (Figure 3).

The source of *Lactobacillus* in the uterus is easily explained by the abundance of this bacterial genus in the nearby vagina (although again the *Lactobacillus* species could differ). It is also possible that *Lactobacillus* reported in many uterine reports is a result of contamination from the vagina. The presence of other taxa that, in some cases, constitute a significant portion of the uterine microbiome (Table 1) may result from other routes of seeding outlined in Figure 2 (57).

Franasiak et al. investigated the uterine microbiome at the time of IVF and embryo transfer and found that *Flavobacterium* comprised one of the two most abundant taxa of the uterine microbiome (48). *Flavobacterium* was not found in any of the other nine NGS sequencing papers to date. This is particularly surprising due to the prevalence of *Flavobacterium* reported by the study in both ongoing and non-ongoing pregnancy. In addition, the study was unique from other studies in the sequencing methodology employed (The Ion 16S Metagenomics Kit rather than Illumina sequencing or pyrosequencing). Furthermore, *Flavobacterium* has been shown to be a common contaminant in reagents by Salter et al. and Laurence et al., specifically in ultrapure water (35, 47–52, 58, 59). However, it is worth noting that Franasiak et al. included a positive (*Escherichia coli*) and negative control (reagent only control), unlike many of the studies covered in this review (48).

Additional work was carried out by Tao et al. to assess the limits of accurate detection by NGS on single species and polymicrobial cultures (52). It was shown that bacteria culture lysates above 60 cells had accurate taxonomic identification (52). The authors were confident that this method was sufficient in detecting microbiota at this low level. Furthermore, it was shown that none of the taxa present in the negative control were one of the four bacterial strains used to assess the limits of detection using their method (52).

In addition to variability in methodology, patient populations and controls, the FRT is modulated by circulating sex hormones leading to physiological changes that influence microbiota compositions and *vice versa* (60). It is not clear if the uterine microbiome changes over time or during the menstrual cycle. However, Moreno et al. evaluated IVF catheter tips to assess the uterine microbiome across two different time points (47). One sample was taken at the prereceptive phase and another at the receptive phase of the same menstrual cycle to assess a putative shift in microbiome composition in IVF patients (47). In this single study, the uterine microbiome was similar at these hormonal stages in 9 out of 13 patients sampled, which is similar to the vaginal microbiome in its stability through hormonal stages (47, 61). However, the fact that this study was conducted over a short period of time with a small sample size suggests that these results should be viewed with some caution (47). Given the significant impact of menopause on the vaginal microbiome reported in many other studies it would be important to determine the impact of hormonal fluctuations and therapies on the uterine microbiota throughout a woman’s lifespan, as well as, in the setting of gynecologic cancers (60, 62, 63).

## Tourists/Invaders: Uterine Microbiota Link to Poor Reproductive Outcomes and Endometriosis

A healthy endometrium is the foundation for successful implantation and intrauterine infection has been deemed the cause of many reproductive complications (64). The endocervical barrier, as a means of preventing ascension of bacteria from the vagina, can be breached. Kunz et al. performed a study demonstrating that radioactively labeled macrospheres reached the uterine cavity within minutes of being administered at the external cervical os and documented the mechanism of the uterine peristaltic pump that actively moves vaginal content to the uterus (8). Zervomanolakis et al. extended these findings by demonstrating that particles could ascend through the cervix within minutes during the follicular and luteal phases of the cycle (9), clearly establishing the plausibility of bacterial ascension as a route of seeding of the uterine microbiome (Figure 2) (9). In addition, ART procedures may seed the uterine microbiota and drive adverse reproductive and gynecologic outcomes through modulating the local microenvironment (24). A reduction in clinical pregnancy rates has been shown when bacteria were cultured from the IVF catheter tip during ART procedures (65). Alternatively, it may be the ascension of key bacterial species/taxa that may lead to increased susceptibility to reproductive complications rather than simply bacterial seeding of any taxa. Notably, Swidsinski et al. demonstrated that half of the women presenting with BV had a polymicrobial biofilm adhered to the endometrium (66).

The presence of bacteria in the uterus has been associated with poor reproductive outcomes and endometriosis; however, a cause and effect relationship has not been clearly established. In addition, the association between endometriosis and poor fertility has been well documented (67). Uterine microbiota composition has been shown to be significantly different in women with endometriosis (27). Furthermore, Cicinelli et al. have reported that endometriosis patients treated with antibiotics before implantation had significantly better reproductive outcomes compared with those not treated with antibiotics (31), suggesting that the negative impact of endometriosis on reproductive outcomes may be in part attributable to the presence of uterine bacteria. It would not be surprising that the anatomical and physiological changes elicited by endometriosis would result in a significantly different uterine microbiome composition due to the proximity of endometriotic lesions to the uterus. Endometriosis patients have been shown to exhibit a uterine bacterial composition with low levels of *Lactobacillaceae* species and enrichment of *Streptococcaceae, Staphylococcaceae*, and *Enterobacteriaceae* species relative to healthy controls (53). Conversely, changes in the microbiome may potentially trigger endometriosis through modification of the microenvironment. As highlighted in the review by Sirota et al., inflammation in the uterus due to the presence of bacteria may influence the balance of cytokines needed for successful blastocyst development and implantation (68). Taking this concept a step further, an inflammatory cytokine signature of endometriosis may have a significant impact on the microenvironment and reproductive outcomes (69). Correlations exist between various pro-inflammatory cytokines such as IL-6 (70) and anti-inflammatory cytokines and adverse reproductive conditions such as polycystic ovary syndrome, tubal factor infertility, or infertility of unknown origin (71).

Despite these links between endometriosis and reproductive outcomes, however, there is only one report that demonstrates a statistical difference in microbiome profiles, rather than just presence of bacteria, between successful vs. unsuccessful reproductive outcomes (47) (Table 1). These investigators enrolled a cohort of 35 subjects undergoing IVF. Endometrial samples were collected before blastocyst implantation to assess the uterine microbiota profiles, which were classified as either *Lactobacillus* dominant (LD), defined as consisting of >90% *Lactobacillus* spp., or non-*Lactobacillus* dominant (NLD), consisting of <90% *Lactobacillus* spp. These investigators found a significant difference in the reproductive outcomes between these two groups. Women with an LD uterine microbiome had markedly higher rates of implantation [60.7 vs. 23.1% (*P* = 0.02)], pregnancy [70.6 vs. 33.3% (*P* = 0.03)], ongoing pregnancy [58.8 vs. 13.3% (*P* = 0.02)], and live births [58.8 vs. 6.7% (*P* = 0.002)] compared with those with an NLD uterine microbiome composition (47). Interestingly, germ-free mice have been shown to have reduced reproductive success after embryo transfer compared with conventionally housed mice, suggesting a role for the presence uterine microbiota in pregnancy (72). As mentioned earlier, the association between *non-Lactobacillus* species and adverse reproductive outcome has been demonstrated in the vaginal microbiome (56). Consequently, the association between the uterine microbiome obtained from the IVF catheter tip and reproductive success following IVF may simply be a reflection of the vaginal microbial community (e.g., LD) through cross-contamination and its association with reproductive success.

By contrast, Franasiak et al. found no significant difference in uterine microbiota between groups with non-ongoing vs. ongoing pregnancy (48). Similar to the previous study by Moreno et al., they found *Lactobacillus* to be one of the most abundant taxa, but they also reported that *Lactobacillus* was not significantly different between non-ongoing and ongoing pregnancy groups. As mentioned earlier, they identified *Flavobacterium*, as one of the most abundant taxa, which is inconsistent with the current NGS literature. This as-yet-unresolved discrepancy between the Moreno and Franasiak studies highlights the need for additional studies. Data analysis may also play a role in the disparity between these two studies. For example, if Franasiak et al. had used the same 90% cutoff as Moreno et al. to determine LD, they may have reached significance. Larger sample size and standardized procedures to avoid vaginal cross-contamination as discussed herein will be important aspects of future studies aimed at determining the role of vaginal and uterine microbiota and reproductive success.

## Invaders: Uterine Microbiota in Cancer and Disease

Success in identifying unique species in the uterine microbiome that are associated with a particular disease state could potentially be used as microbial biomarkers for prevention, screening, diagnosis, or even treatment to improve health and reproductive success. Besides reproductive outcomes and endometriosis, uterine colonization with BV-associated bacteria has been hypothesized to promote carcinogenesis through microbiota-mediated pathophysiologic changes in the microenvironment (62, 73). Indeed, the microbiome is suspected of playing a general role in carcinogenesis through stimulating host secreted pro-inflammatory cytokines or growth factors as a result of dysbiosis (74). For example, pelvic inflammatory disease has been shown to increase risk of developing endometrial cancer by 1.89-fold in a nationwide population-based retrospective cohort study (75).

A recent study by Walther-António et al. compared the microbiome at various sites in the FRT in patients with endometrial cancer, endometrial hyperplasia (as a cancer precursor group) and those with benign uterine conditions. These investigators collected uterine, Fallopian, ovarian, and peritoneal samples post-hysterectomy and preoperative vaginal, cervical, urine, and stool samples from patients in these three study groups and reported the FRT sites and stool sampled in the cancer and hyperplasia patients. Using the microbiome results combined with patient demographic data, it was possible to statistically associate the presence of *Atopobium vaginae* and a *Porphyromonas* sp. in the FRT as being associated with cancer (35). Future studies should be geared at better understanding the functional impact of these bacterial species on hallmarks of cancer illustrated in Figure 3.

Microbiota can drive cancer through numerous mechanisms including preventing apoptosis, stimulating proliferation, and driving genomic instability that are hallmarks of cancer highlighted in Figure 3 (76). The relationship between these taxa and disease may not be limited to resulting inflammation and secretion of cytokines by the host cells, but may also be influenced by the hormonal status of the host. In particular, sex hormones such as estrogens, which are key drivers in certain cancers, have been implicated in carcinogenesis, raising the question of whether estrogens might influence the microbiomes of the uterus similar to the vagina. Use of the gonadotropin-releasing hormone agonist is associated with a shift in composition of the uterine microbiome, demonstrating the uterine microbiome may be hormonally regulated (53). Gut microbiota have been shown to facilitate the reuptake of estrogen to contribute to the progression of estrogen-driven cancer (77). In support of this notion, it has been reported that both gut microbiota composition and systemic estrogen levels are significantly different in patients with breast cancer compared with healthy patients (78–82). In addition, levels of free estrogens have been shown to be modulated by gut bacteria, through the “estrobolome,” *via* secretion of β-glucuronidase, which deconjugates estrogens into their active metabolites (79, 83). However, studies directed at investigation of the relationship between hormonal status and uterine microbiome composition are still lacking.

In addition to women with endometrial cancers, differences between microbiome profiles in “healthy” (albeit underlying conditions, see Table 1) women vs. those with EP and chronic endometritis (EP + CE) have also been reported (see Figure 3). As previously mentioned, Fang et al. divided women into healthy, EP only and EP + CE groups (see also Table 1) and analyzed samples of both vaginal and uterine microbiota (49). These investigators reported that, compared with samples from healthy subjects, EP or EP + CE samples contained higher proportions of Firmicutes at the phylum level and *Lactobacillus, Gardnerella, Bifidobacterium, Streptococcus*, and *Alteromonas* at the genus level, and confirmed that these differences were statistically significant using AMOVA and ANOSIM analyses (49). The finding that *Lactobacillus* was over three times more abundant in the uterine microbiome of both diseased groups EP and EP + CE, compared with healthy controls may suggest ascension of vaginal bacteria (49). We hypothesize that the cervical barrier may have been disrupted in these disease states, which allowed the ascension of the dominant vaginal bacteria, *Lactobacillus*, into the upper FRT. The question of whether bacterial ascension is causative of EP or whether EP results in the increased cervical permeability and ascension could potentially be addressed by analysis of samples collected longitudinally to determine the timing of *Lactobacillus* expansion in the uterus. This strategy could also address whether there may be a positive feedback loop whereby increased cervical permeability leads to increased colonization of vaginal bacteria in the uterus. Another next step would be to determine the functional impact of specific organisms or groups of organisms on the host epithelium using robust human model systems (54, 55, 84).

High vaginal pH (an indicator of vaginal dysbiosis) was also significantly associated with endometrial cancer in the Walther-António study (35). However, vaginal pH, as a single variable, was not significantly different in the benign group compared with the hyperplasia group. A limitation of this study is that the overall microbiota community structure was not fully reported; thus, any firm relationships between microbiome composition, pH, and cancer remain unclear and require further investigation. Indeed, it would be worthwhile to assess *Lactobacillus* spp. in patients to determine whether low *Lactobacillus* abundance correlates with high pH as previously reported (85), or whether it is some other factor within the tumor microenvironment that increases vaginal pH. Interestingly, increased vaginal pH has also been shown to be associated with endometriosis and GnRHa therapy (86), which may suggest a relationship between the vaginal microenvironment on proliferative uterine diseases driven by hormones or *vice versa*.

## Mucosal Axes and The Endometrial Microenvironment

This review mainly focuses on the FRT microbiota; however, it is important to consider that the uterine microbiome may be impacted by, or exert impact on, other distal mucosal sites, which extend beyond the spatial relationship between the vagina and uterus (Box 1).

Box 1 **Future areas of study and clinical implications of uterine microbiome research.**

- Mucosal axes: is there interplay between mucosal sites through translocation of bacteria, metabolites, and/or inflammation?
- Are uterine/vaginal microbiome compositions, particular species or the metabolome they produce associated with carcinogenesis throughout the female reproductive tract?
- Does the estrobolome influence the uterine microbiome or *vice versa*?
- What is the functional relevance of bacterial tourists/invaders and what is the impact on host physiology and disease pathogenesis?
- Can the microbiome serve as a marker for fertility?
- Does the uterine microbiota change antepartum or postpartum or with parity?
- Does the uterine microbiota vary according to race/ethnicity or other genetic factors?
- Can the introduction of pre- or probiotics alleviate gynecologic conditions or decrease risk of poor gynecologic health outcomes?
- How do specific uterine bacterial species impact epithelial barrier function, host signaling and inflammation?

For example, women with endometriosis show significantly lower levels of *Lactobacillaceae* in the uterine microbiome when undergoing treatment with GnRHa, compared with GnRHa-untreated women (53). Women with endometriosis have also been shown to exhibit increased levels of the pro-inflammatory cytokine IL-6 in follicular fluid, with implications for reproductive function (70). It may be this altered inflammatory profile that drives the uterine microbiota composition seen in women with endometriosis or *vice versa* (53). Production of pre-IL-1β in patients with endometriosis has also been found to induce inflammation in the peritoneum (87). Another study that provides evidence of endometriosis impacting inflammation-linked sequelae is a nationwide Danish study, which assessed 37,661 women hospitalized with endometriosis. The results showed that women, after developing endometriosis, were significantly more likely to develop inflammatory bowel disease, ulcerative colitis or Crohn’s disease, compared with controls (88). Even 20 years after initial hospitalization with endometriosis, these patients had an increased risk of developing ulcerative colitis and Crohn’s disease, underscoring the importance of a better understanding of these complex relationships between mucosal sites (88).

Beyond an inflammatory milieu, we further hypothesize that, similar to immune mediators that communicate through the common mucosal immune system, the mucosal sites of the body interact through exchange of bacteria, metabolites or immune signaling between sites and that dysbiosis at one site could impact the mucosal immune environment at another site (Figure 2). Evidence for this concept includes the following studies. First, using culture-dependent methods, it has been shown that Rhesus monkeys with endometriosis exhibited significantly different proportions of *Lactobacillus* spp. and aerobic and facultative anaerobic Gram-negative bacteria in the intestinal microbiota compared with healthy controls (89). A second study supporting the transfer of microbiota from one mucosal site to another is provided by Fardini et al., who reported transmission of bacteria from the oral microbiome to the placenta in mice (18) (presumably *via* the maternal circulation) as a potential cause of intrauterine infection. Further evidence of translocation of viable bacteria is provided in the review by Potgieter et al. that postulates the translocation of dormant but viable bacteria through the blood (90). These authors further determined that injection of saliva and gingival plaque samples into the tail veins of pregnant mice, to mimic the bacteremia of an oral infection, resulted in species-specific colonization of the placenta by such species as *Neisseria flavescens* or *Neisseria subflava* normally found in oral flora (18). The association between subgingival plaque bacteria and placental bacteria has been demonstrated through comparing hypertensive to normotensive individuals (91). Periodontal pathogens are more abundant in subgingival plaques and the placenta in hypertensive women (91). It has also been shown that the detection of *Gardnerella* or *Ureaplasma* in the vaginal microbiome is associated with preterm labor, which is often associated with intrauterine-infection-driven preterm birth following ascension of these vaginal microbiota (45). However, it was questioned as to whether the preterm labor was induced by microbial risk factors of intrauterine infection since none of the women in the study had documented intra-amniotic infection, therefore suggesting an inflammation-related preterm birth (92). As the authors point out, however, ascending infection and documented intra-amniotic infection is not the only possible mechanism microbiota-related risk of preterm birth and this may also be related to inflammatory factors (93). Bacterial translocation to the uterus through the vasculature has also been demonstrated in *Fusobacterium nucleatum* (Figure 2) (17). However, critically, *F*. *nucleatum* did not persist in the liver and spleen, declining in abundance as time progressed (17). This contrasts with the placenta in which bacterial load increased with time (17). Specificity of *F*. *nucleatum* to the uterine cavity is evidence of the plausibility of the transport of bacteria from the blood to the uterus (17). Furthermore, this specificity also suggests that the uterine microenvironment provides a uniquely favorable niche for certain bacterial taxa. However, hematogenous spread of bacteria has strong critics whom refute the plausibility of the spread of bacteria through the body (32).

In addition to the microbiome and immune environment, metabolites produced by microbiota can also interact with host cells to have a positive or negative impact on the host (Figure 3). An example of a mutually beneficial relationship is the production of vitamins and SCFAs that are produced by the gut microbiome and can act not only as nutrients for cells but may also elicit beneficial epigenetic changes in the host as well as (in the case of propionate and acetate) serving as important satiety signal (94). Many other examples exist and a full description would be beyond the scope of this review (95). Conversely, metabolites produced by an unfavorable microbiome (or diet) may have negative impact on the host (Figure 3).

## Further Areas of Study

Microbiota interactions with the host endometrial microenvironment will be an important area of research as we continue to elucidate potential mechanisms that drive disease in the uterus as well as reproductive outcome (69). The uterine microbiota composition may have unique consequences for the endometrial microenvironment due to the site-specific differences in anatomical and physiologic features throughout the FRT (2). We and others have shown the site-specific differences in host responsiveness to microbial products and bacteria throughout the FRT epithelia (2, 55, 84, 96, 97). Various models can effectively recapitulate the complex microenvironment of the FRT and have shown utility to understand host–microbiota interactions (98). For example, Laniewski et al. established and characterized a novel 3-D endometrial epithelial model to better understand host–microbiota interactions at this site (55). Assessing the impact of multiple bacterial species using synthetic combinations or patient-derived samples, to mimic the complex uterine microbiota, may help elucidate the host immune mechanisms in response to microbiota at this site (Box 1).

Similarities may exist between the host response mechanisms of the uterine microenvironment and other sites in the FRT (Box 1). For example, vaginal LB lower the pH of the vaginal microenvironment, which inhibits the colonization of dysbiotic species (85, 99). However, it is unclear how the pH of the intrauterine environment is altered by the presence of microbiota. While one study investigating the role of endometrial pH on reproductive outcome did not demonstrate a significant association with *Lactobacillus* abundance and low pH, this could be due to the relative levels of LB required to lower the pH of the endometrium or other biochemical mechanisms.

The physiological pH is an understudied aspect of the uterine microenvironment, which is likely to be influenced by the presence of and composition of microbiota. The influence of the vaginal microbiome on vaginal pH is well documented and profound (85). The uterine pH may have a similarly important association with particular microbiome compositions. It is even plausible that the vaginal pH may impact uterine pH through direct or indirect mechanisms. In the vaginal microbiome, LB play a crucial role in the modulation of pH through their production of lactic acid (85). In lieu of the consistent finding of LB in the uterine microbiome, important questions are raised as to whether uterine pH is altered by LB presence. However, based on decades of research it is unlikely that LB are found at high enough levels to maintain an acidic environment in the uterus; however, a lower physiological pH at this site may result in damage. The limited data currently available concerning uterine pH suggest that it resides at ~pH 7 (47, 100). There is a discrepancy between studies with it being reported that the uterine pH never exceeds 7.2 (100); however, Moreno et al. reported a range of 6.6–8.51 uterine pH across different patients. Further research is needed to define “normal” uterine pH as the implications of this finding may extend to fertility as well as reproductive and gynecologic sequelae. For example, the neonatal Fc receptor (FcRn), which plays a key role in trafficking immunoglobulins across mucosal tissues, including the uterus has been shown to be pH sensitive. At pH of 6–6.5 the receptor is functional; however at pH of 7, it is non-functional and inhibits transport of IgG, which has significant implications for sexually transmitted infections such as *Chlamydia trachomatis* as it has been shown that IgG translocation *via* FcRn significantly reduces infection (101). Moreno et al. found no association with pH and uterine LB dominance or reproductive success (47). However, additional research is needed in this area to better understand the physiological impact of the presence of uterine bacteria on uterine pH and the local microenvironment (Figure 3).

Future studies should aim at studying the functional relevance of the presence of microbiota in the uterine cavity in terms of pathophysiological mechanisms that contribute to disease pathogenesis (mechanisms outlined in Figure 3). Longitudinal studies assessing the stability of uterine microbiota could help discern whether bacteria colonize transiently (tourists/invaders) or whether there is a stable population (residents). Ascertaining the viability of bacteria will also aid in distinguishing whether microbiota are truly residents of the uterus that contribute to homeostasis or represent microbial DNA left behind from previous transient bacterial tourists or invaders.

## Conclusion

Based on the current literature evaluated in this review, the evidence for a “core” or bacterial resident population in the uterus is lacking and therefore the presence of uterine microbiota are likely reflective of bacterial tourists or invaders rather than a resident population that contributes to health and homeostasis. Uterine microbiota and specific bacterial species may be linked to critical health issues such as endometriosis, endometrial cancer and rates of IVF success. Public health programs will benefit from expanded studies of host–microbiota and host–metabolome interactions within the FRT (summarized in Figure 3). For optimal success, future studies require well-designed and larger patient cohorts to elucidate interactions between the uterine microbiota and host in the context of women’s health. Specific species or microbiota compositions may provide indicators or predictors of disease, participate as mere passengers or act as microbial drivers of disease. As evidence for interactions between the microbiome at mucosal sites increases, other diseases of dysbiosis may drive poor reproductive and gynecologic health outcomes by impacting the uterine microbiota. Studies are needed to further investigate if a “core” or resident uterine microbiota exists and the contributions to health and homeostasis. Furthermore, additional research is warranted to elucidate the functional impact of uterine microbiota or specific bacterial species that may participate as tourists or microbial invaders of this mucosal site and the impact these microbes have on the physiology of the local endometrial microenvironment.

## Author Contributions

MH-K designed the scope and organization of the review and supervised writing. JB and MH-K conducted literature reviews, figure, and table construction and contributed to the writing of the manuscript. DC and MH-K critically edited and reviewed the complete manuscript, tables and figures. All authors approved the final manuscript for submission.

## Conflict of Interest Statement

The authors declare that the research was conducted in the absence of any commercial or financial relationships that could be construed as a potential conflict of interest.
